# Leading the Transformation of Saudi Arabia's Health System Under Vision 2030: Mediating Pathway From Transformational Leadership to Job Performance via Work Engagement Among Nursing Workforce

**DOI:** 10.1002/nop2.70767

**Published:** 2026-08-26

**Authors:** Ibrahim Abdullatif Ibrahim, Abdulaziz F. Abaoud, Sahar Hassan Helaly, Fahad M. Alhowaymel, Atallah Alenezi, Abdullah Abdulrahman Alhomeed, Hala Gaber Elatroush, Mennat Allah G. Abou Zeid

**Affiliations:** ^1^ Department of Nursing Sciences College of Applied Medical Sciences, Shaqra University Shaqra Saudi Arabia; ^2^ Department of Nursing Administration, Faculty of Nursing Mansoura University Mansoura Egypt; ^3^ Department of Nursing Sciences College of Applied Medical Sciences, Taibah University Al Ula Saudi Arabia; ^4^ College of Nursing, Imam Mohammad Ibn Saud Islamic University (IMSIU) Riyadh Saudi Arabia; ^5^ Academic Affairs, Training, and Research, Shaqra Sector Ministry of Health Shaqra Saudi Arabia; ^6^ Department of Nursing Administration and Education College of Nursing, Prince Sattam Bin Abdulaziz University Al‐Kharj Saudi Arabia; ^7^ Department of Nursing Administration, Faculty of Nursing Ain Shams University Cairo Egypt

**Keywords:** job performance, nursing workforce, Saudi Arabia, transformational leadership, vision 2030, work engagement

## Abstract

**Aim:**

To examine the mediating role of work engagement in the relationship between transformational leadership and job performance among nurses in Saudi Arabia.

**Design:**

A multicenter cross‐sectional study.

**Methods:**

This study included a convenience sample of 321 nurses recruited from five tertiary hospitals selected from the Second and Third Health Clusters in Riyadh, Saudi Arabia. Data were collected using a structured web‐based survey incorporating the Global Transformational Leadership Scale, the Utrecht Work Engagement Scale, the Job Performance Scale, and a demographic and professional characteristics questionnaire. Data were analysed using descriptive and inferential statistics in IBM SPSS Statistics (version 27), followed by mediation analysis using Hayes' PROCESS Macro (Model 4).

**Results:**

Nurses reported relatively high perceptions of transformational leadership, relatively high levels of work engagement and high levels of job performance. Transformational leadership was positively associated with work engagement, while gender and education were not significantly associated with work engagement. Transformational leadership and work engagement were positively associated with job performance. Work engagement partially mediated the association between transformational leadership and job performance.

**Conclusion:**

Transformational leadership contributes to higher nurse job performance through both direct effects and by enhancing work engagement. Leadership development and engagement‐focused strategies are essential to strengthen workforce effectiveness.

**Implications for the Profession and/or Patient Care:**

These findings provide evidence to inform leadership training and workforce management policies in healthcare organizations, emphasizing engagement as a mechanism to optimize nurses' performance and indirectly improve patient care outcomes.

**Impact:**

This study addresses the need for effective leadership to strengthen nursing workforce performance within healthcare transformation initiatives. Findings provide actionable insights for healthcare managers and policymakers seeking to implement leadership and engagement strategies that improve organizational outcomes and patient care in Saudi Arabia.

**Reporting Method:**

This study was reported in accordance with the Strengthening the Reporting of Observational Studies in Epidemiology guidelines.

**Patient or Public Contribution:**

No patient or public contribution.

## 1 Introduction

Health systems worldwide are increasingly challenged by workforce shortages, rising patient acuity, rapid technological advancements and growing expectations for quality and safety (Glarcher et al. 2025). These challenges are particularly evident in nursing, where workforce performance and engagement play a critical role in determining patient outcomes, organizational effectiveness and overall system sustainability (Parr et al. 2021; Wei et al. 2023; Slåtten et al. 2022). In Saudi Arabia, these issues intersect with the strategic priorities of Vision 2030, a national reform agenda that emphasizes human capital development, service excellence and the modernization of healthcare delivery (Al‐Hanawi et al. 2019; Alasiri and Mohammed 2022). Achieving these goals requires leadership approaches capable of effectively supporting and motivating the nursing workforce within a rapidly evolving healthcare landscape.

Leadership is widely recognized as a key determinant of work environment quality and staff performance in healthcare settings (Zhao et al. 2024; Xue et al. 2025). Among various leadership styles, transformational leadership has received considerable attention due to its consistent association with positive organizational and individual outcomes. Characterized by inspirational motivation, idealized influence, individualized consideration and intellectual stimulation, transformational leadership has been linked to improved job satisfaction, stronger organizational commitment, reduced burnout, and enhanced job performance among nurses (Boamah et al. 2018; Lai et al. 2020; Gebreheat et al. 2023; Ibrahim et al. 2023; Specchia et al. 2021). By fostering supportive and empowering work environments, transformational leaders encourage professional development, adaptability and collaborative practice (Iqbal et al. 2020; Quesado et al. 2022; Zhang, Chen, et al. 2022). In the Saudi context, the relevance of transformational leadership is further amplified by distinctive workforce characteristics, including a multicultural and predominantly expatriate nursing workforce, hierarchical organizational structures, and, in certain settings, gender‐segregated work environments. These contextual factors may influence how leadership behaviours are perceived and how motivational processes are activated, underscoring the importance of examining not only direct effects but also the mechanisms through which leadership impacts performance.

Work engagement has emerged as a central construct in organizational and nursing research. It is defined as a positive, fulfilling psychological state characterized by vigour, dedication and absorption (Schaufeli et al. 2006), reflecting the extent to which individuals invest their physical, cognitive and emotional energies in their work roles. In nursing settings, higher levels of work engagement have been associated with improved patient safety behaviours, better quality of care, reduced turnover intentions, and enhanced job performance (Slåtten et al. 2022; Huang et al. 2025). As such, work engagement represents a key pathway through which organizational factors, including leadership, may translate into improved performance outcomes.

The Job Demands–Resources (JD–R) model provides a well‐established theoretical framework for understanding these relationships (Bakker and Demerouti 2007). The model distinguishes between job demands, which require sustained physical or psychological effort and job resources, which help employees achieve work goals, reduce job demands, and stimulate personal growth and motivation. In nursing, relevant job resources may include supportive leadership, autonomy, feedback, opportunities for professional development, adequate staffing and resources, social support from colleagues, and opportunities for participation in decision‐making. Within this framework, transformational leadership represents one important job resource that may strengthen nurses' motivation, psychological resources and work engagement. However, it is not the only potential resource influencing engagement and performance. Other organizational and interpersonal resources may also contribute to these outcomes and may partly explain why leadership does not account for all variation in nurses' performance. In this study, transformational leadership was examined as the primary job resource, while recognizing that other job resources may operate concurrently within the nursing work environment. This perspective provides a theoretically grounded basis for examining work engagement as a mechanism linking transformational leadership with job performance.

Although previous studies in Saudi Arabia have demonstrated associations between transformational leadership, work engagement and various nursing outcomes, the existing evidence remains fragmented. Saudi studies have primarily examined leadership in relation to job satisfaction, organizational commitment, innovative behaviour, intention to stay or work engagement, while other studies have investigated work engagement as an outcome of leadership or as a predictor of selected work outcomes (AL‐Dossary 2022; Alharbi et al. 2021; Adalin et al. 2025). Much of the existing empirical evidence has been generated in non‐Saudi contexts, including China, Portugal, Taiwan and Finland (Lai et al. 2020; Huang et al. 2025; Carless et al. 2000; van Beveren et al. 2017), where differences in organizational culture, workforce composition and leadership practices may limit the applicability of findings to the Saudi healthcare system. However, limited research has simultaneously examined transformational leadership, work engagement and job performance within a single theoretically grounded model among nurses in Saudi Arabia. Moreover, the ongoing transformation of the Saudi healthcare system under Vision 2030 provides a distinctive context in which leadership practices and workforce expectations are evolving rapidly. Therefore, findings from other healthcare systems may not fully capture the mechanisms through which leadership influences nurses' performance within the Saudi context.

**FIGURE 1:**
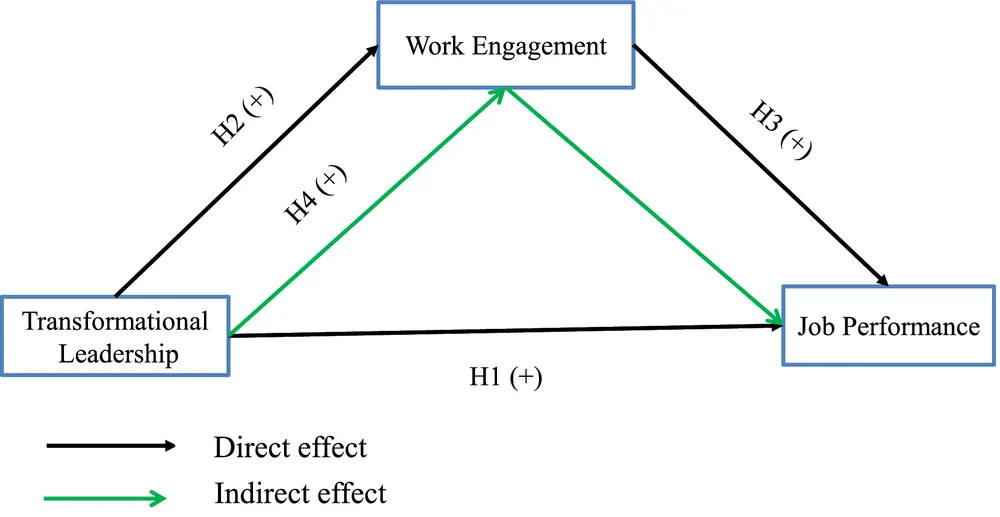
The conceptual model of the study.

To address these gaps, this study investigates the relationship between transformational leadership and job performance among nurses in Saudi Arabia, with particular emphasis on the mediating role of work engagement. Unlike previous Saudi studies that have largely examined these constructs separately or focused on alternative outcomes, this study integrates transformational leadership, work engagement and job performance within a single, theory‐driven mediation model based on the JD‐R framework. This approach is particularly relevant in the Saudi healthcare context, where Vision 2030 is driving substantial organizational, technological and workforce transformation. Understanding whether and how transformational leadership translates into improved performance through nurses' engagement may provide context‐specific evidence to inform leadership development and workforce strategies during this transformation. The study therefore contributes to the literature by examining not simply whether transformational leadership is associated with performance, but also by clarifying the psychological mechanism through which this relationship may occur among nurses in Saudi Arabia. The following hypotheses are proposed and presented in Figure 1:

*Transformational leadership is positively associated with job performance among nurses*.

*Transformational leadership is positively associated with work engagement among nurses*.

*Work engagement is positively associated with job performance among nurses*.

*Work engagement mediates the relationship between transformational leadership and job performance among nurses*.

## 2 Methods

Design: A multicenter cross‐sectional study was utilized.

### 2.1 Setting and Participants

This study was carried out across five tertiary hospitals in Saudi Arabia. The participating hospitals were selected from the Second and Third Health Clusters through a simple ballot procedure, whereby all eligible hospitals had an equal chance of being chosen. Following hospital selection, 321 nurses were recruited using convenience sampling.

Participants were eligible if they were licensed nurses working full‐time and had completed at least 1 year of clinical practice. The minimum experience criterion was established to ensure that participants had adequate exposure to the clinical work setting and relevant leadership practices, thereby enabling them to provide informed assessments of the study variables. Nurses with less than 1 year of clinical experience may still be undergoing professional socialization and adjustment to their roles, with comparatively limited integration into the clinical environment. Including such participants could therefore introduce variability associated with early‐career transition rather than the constructs examined in this study. Healthcare professionals from disciplines other than nursing and nursing interns were not eligible for participation. The study was reported in accordance with the Strengthening the Reporting of Observational Studies in Epidemiology (STROBE) guidelines, supporting transparent reporting and methodological rigour.

### 2.2 Sample Size Calculation

A priori sample size calculation was conducted using G*Power (Version 3.1.9.7). The analysis was based on a linear multiple regression model with a fixed model and an *R* ^2^ deviation from zero, incorporating two predictors: transformational leadership and work engagement. Assuming a medium effect size (*f* ^2^ = 0.15), a two‐sided significance threshold of *α* = 0.05, and a statistical power of 95% (1 − *β* = 0.95), the analysis yielded a minimum required sample of 107 participants.

To enhance participant recruitment and allow for non‐response and eligibility‐related exclusions, 350 nurses from the participating hospitals were approached. Of these, 321 eligible nurses completed the questionnaire and were retained for the final analysis, corresponding to a completion rate of 90.7%. Seventeen eligible nurses chose not to participate, whereas 12 individuals who were approached were excluded because they did not satisfy the predefined eligibility criteria.

### 2.3 Data Collection Procedures

Data collection took place from July to October 2025 through a structured online questionnaire administered via Google Forms. The survey invitation and access link were distributed through official institutional email accounts, providing a controlled mechanism for accessing the questionnaire and verifying participants' institutional identity. To encourage participation, a follow‐up reminder was issued 2 weeks after the initial invitation. Before accessing the questionnaire, potential participants were presented with an electronic informed consent form that explained the study's purpose, procedures, and participants' rights. Participation was confirmed by selecting the ‘I agree’ option, whereas those who did not provide consent were directed automatically to an exit page and could not proceed to the survey. Several measures were implemented to promote data quality and completeness. Institutional email authentication was used to restrict participation to one response per participant, thereby preventing duplicate submissions. In addition, all questionnaire items were configured as mandatory fields, ensuring that completed responses contained no missing data.

### 2.4 Measures and the Study Variables

#### 2.4.1 Transformational Leadership (Independent Variable)

The Global Transformational Leadership Scale (GTLS) was employed to measure nurses' perceptions of transformational leadership behaviours demonstrated by their immediate nurse managers (Carless et al. 2000). The GTLS was selected because it provides a concise measure of core transformational leadership behaviours and has demonstrated satisfactory psychometric properties in previous studies (Ibrahim et al. 2023; van Beveren et al. 2017). The GTLS consists of seven items, each rated on a five‐point Likert response format ranging from 1 (‘strongly disagree’) to 5 (‘strongly agree’). In this study, the reliability of the GTLS was 0.89, indicating good internal consistency. Confirmatory factor analysis (CFA) was conducted using AMOS version 24 with the final sample of 321 nurses to evaluate the hypothesized one‐factor structure of the seven‐item GTLS. The confirmatory factor analysis (CFA) for GTLS The model demonstrated acceptable fit to the data (Chi‐square (*χ* ^2^), degrees of freedom (df), *χ* ^2^/df = 2.91), (comparative fit index (CFI) = 0.996), (Tucker–Lewis index (TLI) = 0.969), (root mean square error of approximation (RMSEA) = 0.077), and (standardized root mean square residual (SRMR) = 0.022).

#### 2.4.2 Work Engagement (Mediating Variable)

Work engagement was assessed using the nine‐item short version of the Utrecht Work Engagement Scale (UWES‐9) (Schaufeli et al. 2006). The scale comprises three dimensions: vigour, dedication and absorption. Items are rated on a seven‐point scale ranging from 0 (‘never’) to 6 (‘always’), with higher scores indicating greater work engagement. In this study, the UWES demonstrated excellent internal consistency (Cronbach's *α* = 0.92), while Cronbach's *α* values for vigour, dedication and absorption were 0.83, 0.79 and 0.81, respectively. CFA was conducted using AMOS version 24 with the final sample of 321 nurses to evaluate the hypothesized three‐factor model comprising vigour, dedication, and absorption. The model demonstrated good fit (*χ* ^2^/df = 1.44, CFI = 0.994, TLI = 0.990, RMSEA = 0.037, and SRMR = 0.021).

#### 2.4.3 Job Performance (Dependent Variable)

The Job Performance Scale (JPS) developed by Ke et al. (2022), was used to assess nurses' job performance. The JPS includes two dimensions: task performance (4 items) and contextual performance (10 items). Respondents rated each item on a five‐point Likert scale ranging from 1 (‘never’) to 5 (‘always’), with higher scores reflecting higher levels of job performance. In this study, the reliability of the JPS was 0.90, indicating excellent internal consistency. The subscales also demonstrated strong reliability, with Cronbach's alpha values of 0.80 for task performance and 0.87 for contextual performance. CFA was conducted using AMOS version 24 with the final sample of 321 nurses to evaluate the hypothesized two‐factor measurement model comprising task and contextual performance. The model demonstrated acceptable fit (*χ* ^2^/df = 2.98, CFI = 0.959, TLI = 0.918, RMSEA = 0.079 and SRMR = 0.046).

#### 2.4.4 Demographics and Professional Characteristics (Controlling Variables)

The demographic characteristics questionnaire includes questions about the participants' age, gender, education, marital status, experience years, nationality, working hours, nursing job and education.

The standardized instruments were translated from English into Arabic and subsequently back‐translated in accordance with Brislin's guidelines to ensure linguistic equivalence and cultural appropriateness (Beaton et al. 2000). To assess the face and content validity of the Arabic versions, five professors specializing in nursing management independently reviewed the translated instruments. The Scale Content Validity Index (S‐CVI) values were 0.90 for the GTLS, 0.93 for the UWES, and 0.91 for the JPS, demonstrating a high level of content validity for all three measures (Polit and Beck 2006). A pilot study involving 23 nurses was subsequently conducted to assess the clarity, comprehensibility and practical applicability of the instruments. The pilot participants reported no difficulties or concerns regarding the questionnaire items, supporting the clarity and suitability of the measures for the main study. Data obtained from the pilot sample were not included in the final statistical analysis.

### 2.5 Statistical Analysis

Data analysis was conducted using IBM SPSS Statistics version 27 and AMOS version 24. The distributional properties of the data were assessed using multiple approaches. The Central Limit Theorem was considered in interpreting the approximate normality of the sampling distribution, given the sample size exceeded 30 participants (Field 2024). In addition, histograms were examined for evidence of substantial departures from normality, while skewness and kurtosis were evaluated using their corresponding *z*‐scores. The absolute *z*‐scores were within the predefined acceptable ranges, and visual inspection revealed no marked deviations from a normal distribution, supporting the assumption of approximate normality (Kim 2013).

Descriptive analyses were performed to characterize the participants and summarize the principal study variables. Continuous variables were presented as means and standard deviations, whereas categorical variables were described using frequencies and percentages. Differences in study variables across categorical demographic characteristics were examined using independent‐samples *t*‐tests or one‐way analysis of variance (ANOVA), as appropriate. Pearson's correlation coefficients were subsequently calculated to assess the direction and strength of associations among the principal study variables.

The hypothesized mediating relationship was examined using Hayes' PROCESS macro (version 4.2 beta, Model 4). The indirect effect was estimated using 5000 bias‐corrected bootstrap resamples and was considered statistically significant when its 95% confidence interval did not contain zero. Demographic characteristics that demonstrated statistically significant associations with the study variables were initially considered for inclusion as covariates. Following this assessment, gender and educational level were retained in the final mediation model because they demonstrated consistent and statistically significant associations with the variables included in the analysis.

Confirmatory factor analysis (CFA) was performed using AMOS to evaluate the construct validity of the measurement model. Model adequacy was assessed according to the fit criteria proposed by Hu and Bentler, with acceptable model fit defined as *χ* ^2^/df < 5, comparative fit index (CFI) > 0.90, Tucker–Lewis index (TLI) > 0.90, root mean square error of approximation (RMSEA) < 0.08 and standardized root mean square residual (SRMR) < 0.08 (Hu and Bentler 1999). For all statistical tests, a two‐sided *p* value of < 0.05 was considered indicative of statistical significance.

### 2.6 Ethical Considerations

Ethical approval was granted by the Institutional Research Ethics Committee of Shaqra University (Approval No. ERC_SU_S_202500186). The study was conducted in accordance with the ethical principles outlined in the Declaration of Helsinki. Before accessing the questionnaire, all participants provided electronic informed consent.

The introductory page of the online survey presented participants with clear information regarding the study's purpose and procedures and emphasized that participation was voluntary. Participants were informed that they could withdraw from the study at any point without penalty or adverse consequences. An electronic consent statement was incorporated into the first section of the survey, and access to the questionnaire was permitted only after participants confirmed their willingness to participate.

Participant confidentiality was protected throughout the study. No personally identifiable information was collected, and the survey was designed to preserve participants' anonymity. The collected data were maintained in a secure, password‐protected system, with access restricted to authorized members of the research team. Participation was entirely uncompensated, and no financial or other incentives were provided.

## 3 Results

### 3.1 Participant Characteristics and Variations in Study Variables

The average age of the participants was 34.53 years, with a standard deviation of 7.56 years. A significant portion of the participants (45.2%) were between the ages of 31 and 40. The majority were female (65.4%), married (57.9%), non‐ Saudi (55.8%), working fixed shift (61.4%) and staff nurses (84.4%). Most of the nurses in the study held a bachelor's degree in nursing education (60.4%) and had more than 10 years of experience, which constituted 48.3% of the sample. The mean years of work experience among the participants was 10.99 years, with a standard deviation of 7.64 years (Table 1). Significant differences were found in transformational leadership, work engagement and job performance based on gender group (*p* < 0.01). Additionally, work engagement and job performance varied significantly by education group (*p* < 0.05) (Table 2).

**TABLE 1:** Characteristics of the participants.

Characteristics	Category	*N*	%
Age	20–30	108	33.6
	31–40	145	45.2
	> 40	68	21.2
	Mean (SD)	34.53 (7.56)	
Gender	Male	111	34.6
	Female	210	65.4
	t/p		
Marital status	Unmarried	135	42.1
	Married	186	57.9
Experience	1–5	94	29.3
	6–10	72	22.4
	> 10	155	48.3
	Mean (SD)	10.99 (7.64)	
Nationality	Saudi	142	44.2
	Non‐Saudi	179	55.8
Working hours	Fixed	197	61.4
	Rotated	124	38.6
Nursing job	Staff nurse	271	84.4
	Nurse managers	50	15.6
Education	Technical degree	103	32.1
	Bachelor degree	194	60.4
	Postgraduates degree	24	7.5

**TABLE 2:** Mean scores differences in the study variables.

Characteristics	Category	TL	WE	JP
		Mean (SD)	Mean (SD)	Mean (SD)
Age	20–30	3.85 ± 1.05	3.90 ± 1.50	4.00 ± 0.81
	31–40	3.84 ± 0.92	3.94 ± 1.41	4.10 ± 0.75
	> 40	4.03 ± 0.86	4.34 ± 1.43	4.28 ± 0.69
	f/p	0.948/0.389	2.229/0.109	2.830/0.061
Gender	Male	3.71 ± 0.97	3.72 ± 1.42	3.95 ± 0.76
	Female	3.98 ± 0.94	4.17 ± 1.44	4.19 ± 0.75
	t/p	2.42/0.016*	2.65/0.009**	2.76/0.006**
Marital status	Unmarried	3.91 ± 1.03	4.04 ± 1.48	4.14 ± 0.77
	Married	3.87 ± 0.90	3.99 ± 1.43	4.08 ± 0.76
	t/p	0.37/0.711	0.34/0.737	0.70/0.487
Education	Technical degree	3.81 ± 0.97	3.77 ± 1.42	3.95 ± 0.77
	Bachelor degree	3.93 ± 0.96	4.19 ± 1.45	4.20 ± 0.74
	Postgraduates degree	3.88 ± 0.85	3.63 ± 1.41	4.04 ± 0.81
	f/p	0.60/0.551	3.87/0.022*	3.60/0.028*
Experience	1–5	3.83 ± 1.01	3.91 ± 1.45	3.99 ± 0.80
	6–10	3.89 ± 0.97	4.00 ± 1.49	4.17 ± 0.75
	> 10	3.92 ± 0.92	4.08 ± 1.43	4.15 ± 0.75
	f/p	0.28/0.760	0.37/0.691	1.57/0.210
Nationality	Saudi	3.94 ± 1.02	4.00 ± 1.53	4.12 ± 0.81
	Non‐Saudi	3.84 ± 0.90	4.02 ± 1.39	4.10 ± 0.72
	t/p	0.93/0.352	−0.14/0.891	0.29/0.773
Working hours	Fixed	3.88 ± 0.97	4.04 ± 1.43	4.11 ± 0.78
	Rotated	3.90 ± 0.93	3.97 ± 1.49	4.10 ± 0.74
	t/p	0.23/0.819	0.44/0.665	0.02/0.984
Nursing job	Staff nurse	3.93 ± 0.93	4.03 ± 1.42	4.11 ± 0.74
	Nurse managers	3.68 ± 1.08	3.90 ± 1.59	4.06 ± 0.89
	t/p	1.68/0.094	0.60/0.551	0.46/0.644

*Note: t*, independent *t*‐test; *F*, analysis of variance.

Abbreviations: JP, job performance; TL, transformational leadership; WE, work engagement.

* *p* < 0.05.

** *p* < 0.001.

### 3.2 Descriptive and Correlational Statistics of Study Variables

The mean score for transformational leadership was 3.89 (SD = 0.96), indicating relatively favourable perceptions of transformational leadership among participants, with sufficient variability in responses. Overall work engagement had a mean score of 4.01 (SD = 1.45), with the subdimensions of vigour, dedication and absorption ranging from 3.91 (SD = 1.57) to 4.10 (SD = 1.52). Job performance also demonstrated a relatively high mean score of 4.11 (SD = 0.76), with task and contextual performance ranging from 4.10 (SD = 0.82) to 4.15 (SD = 0.80). Although the relatively high mean scores may suggest a potential ceiling effect, the observed standard deviations indicate meaningful variability in participants' responses rather than strong clustering at the upper end of the scales. Thus, the findings appear to reflect generally favourable perceptions of leadership, engagement, and performance while retaining sufficient response variability for subsequent analyses. Transformational leadership was positively and strongly correlated with overall work engagement (*r* = 0.642, *p* < 0.001) and with job performance (*r* = 0.719, *p* < 0.001), including both task (*r* = 0.658, *p* < 0.001) and contextual performance (*r* = 0.673, *p* < 0.001). Work engagement also demonstrated strong positive associations with overall job performance (*r* = 0.767, *p* < 0.001) as well as with task (*r* = 0.573, *p* < 0.001) and contextual performance (*r* = 0.768, *p* < 0.001) (Table 3).

**TABLE 3:** Descriptive statistics and correlation analysis.

The study variables	Mean (SD)	1	2	3	4	5	6	7	8
1. Transformational leadership	3.89 (0.96)	1							
2. Work engagement	4.01 (1.45)	0.642***	1						
3. Vigour	3.91 (1.57)	0.557***	0.908***	1					
4. Dedication	4.05 (1.53)	0.624***	0.917***	0.797***	1				
5. Absorption	4.10 (1.52)	0.650***	0.889***	0.732***	0.768***	1			
6. Job performance	4.11 (0.76)	0.719***	0.767***	0.688***	0.739***	0.708***	1		
7. Task performance	4.15 (0.80)	0.658***	0.573***	0.540***	0.543***	0.562***	0.746***	1	
8. Contextual performance	4.10 (0.82)	0.673***	0.768***	0.691***	0.739***	0.704***	0.923***	0.626***	1

*** *p* < 0.001.

### 3.3 Mediation Outcomes

A mediation analysis demonstrated that transformational leadership was associated positively with work engagement (*β* = 0.632, *t* = 14.595, *p* < 0.001), explaining 41.7% of the variance (*R* ^2^ = 0.417, *F*(3, 317) = 75.629, *p* < 0.001). In contrast, neither gender nor education showed a statistically significant association with work engagement. When predicting job performance, both transformational leadership (*β* = 0.383, *t* = 9.208, *p* < 0.001) and work engagement (*β* = 0.514, *t* = 12.331, *p* < 0.001) emerged as significant positive predictors. The overall model explained a substantial proportion of variance in job performance (*R* ^2^ = 0.680, *F*(4, 316) = 167.662, *p* < 0.001). Gender and education were not significantly associated with job performance. The total effect of transformational leadership on job performance was statistically significant (*β* = 0.708, *t* = 18.117, *p* < 0.001), indicating a strong overall relationship. After including work engagement in the model, the direct effect of transformational leadership on job performance remained significant but was reduced in magnitude (*β* = 0.383, *p* < 0.001), suggesting partial mediation. Furthermore, the indirect effect of transformational leadership on job performance through work engagement was significant (*β* = 0.325, 95% CI [0.257, 0.398]), as the confidence interval did not include zero (Table 4).

**TABLE 4:** Mediation analysis of the effect of transformational leadership on job performance via work engagement.

Outcomes	Predictors/covariates	*β*	*t*	95% CI (LL–UL)	Model summary
WE	TL	0.632	14.595***	0.830–1.089	*R* ^2^ = 0.417; *F*(3, 317) = 75.629***
	Gender	0.063	1.444	0.069–0.449	
	Education	0.032	0.746	0.131–0.291	
JP	TL	0.383	9.208***	0.241–0.371	*R* ^2^ = 0.680; *F*(4, 316) = 167.662***
	WE	0.514	12.331***	0.228‐0.314	
	Gender	0.027	0.845	−0.058‐0.145	
	Education	0.054	1.693	−0–.012‐0.154	
Total effect (*c*)	TL → JP	0.708	18.117***	0.504–0.627	*R* ^2^ = 0.526; *F*(3, 317) = 117.079***
Direct effect (*c*')	TL → JP	0.383	9.208***	0.241–0.371	
Indirect effect	TL → WE → JP	0.325		0.257–0.398	

Abbreviations: JP, job performance; TL, transformational leadership; WE, work engagement.

*** *p* < 0.001.

## 4 Discussion

This study examined the influence of transformational leadership on job performance among nurses in Saudi Arabia, with particular attention to the mediating role of work engagement, in alignment with the health system transformation goals articulated in Vision 2030. The findings demonstrate a positive association between transformational leadership and nurses' job performance, thereby supporting H1. This relationship suggests that when nurses perceive their immediate supervisors as exhibiting transformational leadership behaviours, such leaders may foster greater autonomy, critical thinking and collaboration; these competencies are essential for managing complex patient needs and adapting to rapidly evolving clinical environments. Through these mechanisms, transformational leadership appears to enhance observable indicators of job performance, including quality of care, efficiency and adherence to clinical standards. These results are consistent with prior international research indicating a robust positive relationship between transformational leadership and employee performance (Lai et al. 2020). Empirical evidence from nursing contexts further supports this interpretation; for instance, Boamah et al. (2018) reported that transformational leadership positively influences workplace empowerment, which in turn enhances nurses' performance and job satisfaction. More recent systematic reviews have likewise shown that transformational leadership contributes not only to improved performance but also to proximal outcomes such as job satisfaction, retention intentions and patient safety, suggesting that enhanced performance may emerge within a broader pattern of favourable work environments and professional functioning (Boamah et al. 2018).

The Saudi healthcare context provides an important perspective for interpreting these findings. Under Vision 2030, the health system is shifting towards more integrated, patient‐centred, value‐oriented and digitally enabled care. In this rapidly changing context, transformational leadership extends beyond traditional supervision to support organizational and technological change. Nurse managers can translate transformation priorities into clinical practice, encourage staff participation, and facilitate adaptation to new care models and digital technologies. Through shared vision, intellectual stimulation, and individualized consideration, transformational leaders may help nurses understand and engage with these changes, particularly within a healthcare system undergoing simultaneous organizational, technological and cultural transformation.

Consistent with H2, the results indicate a positive association between transformational leadership and work engagement among nurses. This finding underscores the influential role of leadership in shaping nurses' motivational and psychological states. Leaders who articulate an inspiring vision, demonstrate individualized consideration and encourage innovation appear to cultivate higher levels of vigour, dedication and absorption among nursing staff. The present results align with a growing body of quantitative research conducted across hospital and intensive care settings, which consistently reports significant positive associations between transformational leadership behaviours and nurses' work engagement (Boamah et al. 2018; AL‐Dossary 2022; Zhang et al. 2025; Ystaas et al. 2023; Alluhaybi et al. 2024). These findings also corroborate those of Monje‐Amor et al. (Monje‐Amor et al. 2020), who demonstrated that leadership style, work engagement, and organizational commitment among nurses in Saudi Arabian hospitals. Health care leaders enhance engagement by fostering supportive work climates, strengthening communication, facilitating professional development and ensuring access to adequate resources.

Individualized consideration may be particularly important during healthcare digital transformation. Nurses differ in digital literacy, technology experience and readiness to adopt new systems. Transformational leaders can address these differences through tailored training, mentoring, hands‐on learning and constructive feedback, thereby reducing resistance and strengthening nurses' confidence in using digital technologies. This individualized support may facilitate nurses' adaptation to digital transformation and enhance workforce readiness for the digital healthcare initiatives of Vision 2030.

From a theoretical perspective, the JD–R model provides a useful explanatory framework. According to Schaufeli and Bakker (2004), work engagement comprises vigour, dedication and absorption, reflecting a state in which employees experience their work as meaningful and motivating. The JD–R model posits that engagement increases when job demands are balanced by sufficient resources. Transformational leadership contributes to this balance by augmenting job resources through encouragement, inspiration and individualized support, thereby mitigating the strain of job demands and promoting engagement, performance and well‐being (Hakanen et al. 2018). In the context of Saudi healthcare transformation, these leadership resources may become particularly important as nurses face new technological demands, changing workflows, and evolving expectations for quality and efficiency. Thus, transformational leadership may support engagement not only by motivating nurses but also by helping them access the resources required to navigate complex organizational and technological changes.

In support of H3, work engagement was found to be positively associated with job performance among nurses. This result suggests that engaged nurses are more capable of translating positive work attitudes into sustained, high‐quality performance. The finding is consistent with prior studies emphasizing the central role of engagement in driving task performance in demanding healthcare environments (Ghazawy et al. 2021; Bernales‐Turpo et al. 2022). Collectively, these results indicate that although job satisfaction is an important outcome in its own right, it may be insufficient to ensure optimal performance unless accompanied by high levels of engagement and adequate organizational support.

The mediation analysis further supported H4, demonstrating that work engagement mediates the relationship between transformational leadership and job performance. This finding indicates that work engagement serves as a critical psychological mechanism through which transformational leadership exerts its influence on performance outcomes. While transformational leaders may inspire and motivate nurses through vision, encouragement and individualized consideration, these behaviours appear to enhance performance primarily by fostering higher levels of vigour, dedication and absorption. In line with previous research, studies have shown that transformational leadership influences job performance both directly and indirectly through work engagement and related psychological resources, with engagement accounting for a substantial proportion of the overall effect (Lai et al. 2020; Huang et al. 2025; Kato et al. 2023; Salanova et al. 2011). Conceptually, work engagement represents the psychological energy and commitment that nurses bring to their roles, translating leadership inputs into sustained performance (Zhang, Xu, et al. 2022). By promoting purpose, accountability, and innovative thinking, transformational leaders strengthen nurses' internal resources, enabling them to approach challenges with greater enthusiasm and persistence. When nurses feel valued and supported, their concentration and motivation are enhanced, resulting in higher levels of vigour, dedication and absorption that collectively contribute to improved job performance (Masood and Afsar 2017).

Importantly, gender and education were not significant predictors of nurses' job performance in the final mediation model. This finding suggests that, within this study context, transformational leadership and work engagement may be more proximal influences on nurses' performance than these demographic characteristics. This result is consistent with findings from Iraq, where no significant relationship was observed between nurses' overall job performance and sociodemographic characteristics (Al‐Hasnawi and Adea Aljebory 2023). However, evidence from other settings remains mixed. Research among Iranian nurses identified age, gender, work setting, work shift, clinical experience and psychological frustration as factors associated with job performance (Pourteimour et al. 2021), whereas a study among male nurses in China reported that educational level, length of service, and other professional and personal characteristics were associated with performance (Shen et al. 2022). Similarly, education level has been reported to be associated with nurses' job performance (Sarıköse and Göktepe 2022). More recent evidence from the Philippines found that age and sex significantly predicted nurses' work performance, whereas education was not a significant predictor (May et al. 2026). These inconsistencies may reflect differences in healthcare systems, organizational contexts, workforce characteristics, job demands and performance measurement approaches. In this study, the non‐significant effects of gender and education may therefore indicate that leadership and motivational factors, particularly transformational leadership and work engagement, are more influential in shaping nurses' performance than demographic characteristics. Accordingly, interventions aimed at strengthening transformational leadership and work engagement may be broadly applicable across nurses with different gender and educational backgrounds. Nevertheless, these findings should be interpreted cautiously, as the absence of significant associations may also reflect the characteristics of the study sample or other unmeasured individual, organizational, and contextual factors.

Collectively, these findings suggest that transformational leadership may be particularly valuable in healthcare systems undergoing substantial transformation. In the Saudi context, its contribution may extend beyond improving immediate staff outcomes to supporting nurses as they adapt to value‐oriented care models, digital transformation and evolving organizational expectations. However, given the cross‐sectional nature of this study, these interpretations should be understood as theoretically informed explanations of observed associations rather than evidence of causal effects. Longitudinal and intervention‐based research is warranted to determine whether transformational leadership development, particularly interventions focused on individualized consideration and support for digital adaptation, can strengthen nurses' work engagement and performance during the ongoing transformation of the Saudi healthcare system.

### 4.1 Limitations and Future Research

Several limitations should be considered when interpreting these findings. First, the cross‐sectional design precludes definitive conclusions regarding causality among transformational leadership, work engagement and job performance. Longitudinal or experimental designs are therefore recommended to examine the temporal ordering and stability of these relationships. Second, the use of self‐reported measures may introduce social desirability or common method bias. Although self‐report instruments are widely used in organizational research, future studies could strengthen validity by incorporating objective performance indicators or multi‐source assessments. Third, the use of convenience sampling may have introduced selection bias and limits the external validity of the findings. Accordingly, the results should be interpreted primarily in the context of the accessible nursing population represented by the participating hospitals and should not be generalized to all nurses in the region. Future studies using probability‐based sampling methods are recommended to enhance the representativeness and generalizability of the findings. Fourth, the sample was drawn from a limited number of healthcare institutions within Saudi Arabia, which may constrain the generalizability of the findings to other regions, organizational settings or cultural contexts. Leadership practices and workforce dynamics may vary across healthcare systems, particularly in light of the ongoing structural and digital reforms associated with Vision 2030.

Future research should aim to replicate the proposed mediation model in diverse clinical and cultural settings. Additionally, examining other potential mediators or moderators, such as psychological empowerment, team cohesion or opportunities for professional development, may further elucidate the mechanisms through which transformational leadership influences nurse engagement and performance.

### 4.2 Implications of the Study

The findings have important implications for healthcare leadership, workforce management and health policy within the context of Saudi Arabia's Vision 2030. By identifying a mediated pathway linking transformational leadership to job performance through work engagement, this study highlights leadership as a potentially important organizational strategy for strengthening nursing workforce outcomes. From a policy perspective, the findings support the prioritization of structured leadership development programmes that equip nurse managers with transformational leadership competencies, including inspirational motivation, individualized consideration and intellectual stimulation. Such programmes may enhance nurses' work engagement and performance while also fostering a supportive work environment that encourages professional growth and organizational commitment.

Leadership development may also serve as a significant strategy for nurse retention. Nurse managers who demonstrate transformational leadership behaviours can create a work environment in which nurses feel valued, supported, recognized, and connected to a shared organizational vision. These conditions may strengthen work engagement, job satisfaction and organizational commitment, which are important factors associated with nurses' willingness to remain in their organizations. Therefore, healthcare organizations may consider integrating leadership development with broader nurse retention strategies, including mentoring, career development opportunities, regular leadership training and mechanisms for recognizing nurses' contributions. Such integrated approaches may be particularly relevant in addressing workforce stability and reducing nurse turnover in healthcare systems undergoing substantial transformation.

At the organizational level, nursing leaders are encouraged to adopt emotionally intelligent, supportive and vision‐driven leadership approaches that cultivate engagement and a strong sense of purpose among nursing staff. Higher work engagement may contribute to greater commitment, adaptability and innovation, which are essential for advancing digital transformation, quality improvement and patient‐centred care initiatives under Vision 2030. Hospital administrators may also consider routinely assessing nurses' work engagement and leadership experiences to identify areas for improvement and inform targeted leadership development and workforce retention initiatives. From a research perspective, future longitudinal studies should examine whether leadership development interventions improve work engagement and job performance while also reducing turnover intention and actual nurse turnover. This would provide stronger evidence regarding the role of transformational leadership development as a sustainable strategy for nurse retention.

## 5 Conclusions

This study provides empirical evidence that transformational leadership plays a pivotal role in enhancing nurses' job performance within the framework of Saudi Arabia's Vision 2030 health system transformation. The findings indicate that transformational leadership influences performance both directly and indirectly through work engagement, highlighting the interconnected roles of leadership, motivation and performance in healthcare organizations. Nurses who are more engaged appear better able to translate effective leadership into consistent, high‐quality performance. The results further suggest that fostering transformational leadership behaviours creates an environment in which nurses feel empowered, motivated and fully engaged. Strengthening these leadership capabilities may therefore represent a strategic pathway for developing a resilient, high‐performing nursing workforce capable of meeting the demands of a rapidly evolving healthcare system.

## Author Contributions

**Ibrahim Abdullatif Ibrahim:** conceptualization, methodology, formal analysis, data curation, supervision, writing – review and editing, writing – original draft, validation, visualization. **Abdulaziz F. Abaoud:** investigation, data curation, writing – review and editing. **Sahar Hassan Helaly:** methodology, investigation, writing – review and editing. **Fahad M. Alhowaymel:** investigation, writing – review and editing, data curation. **Atallah Alenezi:** conceptualization, writing – original draft, writing – review and editing. **Abdullah Abdulrahman Alhomeed:** investigation, resources, writing – review and editing. **Hala Gaber Elatroush:** methodology, investigation, writing – review and editing. **Mennat Allah G. Abou Zeid:** methodology, writing – review and editing, supervision, project administration, writing – original draft, conceptualization.

## Funding

The authors have nothing to report.

## Disclosure

Statistics Compliance Statement: The statistics were checked prior to submission by the first author, Ibrahim Abdullatif Ibrahim. The authors affirm that the methods used in the data analyses are suitably applied to their data within their study design and context, and the statistical findings have been implemented and interpreted correctly. The authors agree to take responsibility for ensuring that the choice of statistical approach is appropriate, conducted and interpreted correctly.

## Ethics Statement

Ethical clearance for the study was granted by the Institutional Research Ethics Committee of Shaqra University (Approval No. ERC_SU_S_202500186). Before data collection, all participants provided electronic informed consent after receiving information about the study. Participation was voluntary, and respondents were informed that they could discontinue their participation at any time without any adverse consequences. No personally identifiable information was obtained, and strict measures were implemented to protect participants' privacy and confidentiality. The research data were securely maintained in password‐protected systems, with access restricted to authorized members of the research team.

## Conflicts of Interest

The authors declare no conflicts of interest.

## Data Availability

The data that support the findings of this study are available from the corresponding author upon reasonable request. Data are not publicly available due to participant confidentiality obligations and the absence of explicit participant consent for open data deposition at the time of data collection.
